# Suitability of bilateral filtering for edge-preserving noise reduction in PET

**DOI:** 10.1186/2191-219X-1-23

**Published:** 2011-10-05

**Authors:** Frank Hofheinz, Jens Langner, Bettina Beuthien-Baumann, Liane Oehme, Jörg Steinbach, Jörg Kotzerke, Jörg van den Hoff

**Affiliations:** 1 PET Centre, Institute of Radiopharmacy, Helmholtz-Zentrum Dresden-Rossendorf, Dresden, Germany; 2 Department of Nuclear Medicine, University Hospital Carl Gustav Carus, Technische Universität Dresden, Dresden, Germany

**Keywords:** quantification, PET, SUV_max_, bilateral filtering, spatial resolution, image filtering

## Abstract

**Background:**

To achieve an acceptable signal-to-noise ratio (SNR) in PET images, smoothing filters (SF) are usually employed during or after image reconstruction preventing utilisation of the full intrinsic resolution of the respective scanner. Quite generally Gaussian-shaped moving average filters (MAF) are used for this purpose. A potential alternative to MAF is the group of so-called bilateral filters (BF) which provide a combination of noise reduction and edge preservation thus minimising resolution deterioration of the images. We have investigated the performance of this filter type with respect to improvement of SNR, influence on spatial resolution and for derivation of SUV_max _values in target structures of varying size.

**Methods:**

Data of ten patients with head and neck cancer were evaluated. The patients had been investigated by routine whole body scans (ECAT EXACT HR^+^, Siemens, Erlangen). Tomographic images were reconstructed (OSEM 6i/16s) using a Gaussian filter (full width half maximum (FWHM): Γ_0 _= 4 mm). Image data were then post-processed with a Gaussian MAF (FWHM: Γ_M _= 7 mm) and a Gaussian BF (spatial domain: Γ_S _= 9 mm, intensity domain: Γ_I _= 2.5 SUV), respectively. Images were assessed regarding SNR as well as spatial resolution. Thirty-four lesions (volumes of about 1-100 mL) were analysed with respect to their SUV_max _values in the original as well as in the MAF and BF filtered images.

**Results:**

With the chosen filter parameters both filters improved SNR approximately by a factor of two in comparison to the original data. Spatial resolution was significantly better in the BF-filtered images in comparison to MAF (MAF: 9.5 mm, BF: 6.8 mm). In MAF-filtered data, the SUV_max _was lower by 24.1 ± 9.9% compared to the original data and showed a strong size dependency. In the BF-filtered data, the SUV_max _was lower by 4.6 ± 3.7% and no size effects were observed.

**Conclusion:**

Bilateral filtering allows to increase the SNR of PET image data while preserving spatial resolution and preventing smoothing-induced underestimation of SUV_max _values in small lesions. Bilateral filtering seems a promising and superior alternative to standard smoothing filters.

## Background

Quite often, the practically achievable statistical accuracy of PET data is not sufficient to utilise the full intrinsic spatial resolution of the respective system. Rather, it is usually necessary to perform data smoothing either during image reconstruction or afterwards to achieve a reasonable signal-to-noise ratio (SNR). Typically, Gaussian-shaped moving average filters (MAF) are used for this purpose. While improving the SNR and, therefore, facilitating identification and delineation of sufficiently large low intensity structures, these filters at the same time reduce the spatial resolution of the images, thus deteriorating detectability and quantification accuracy of small lesions. Therefore, the spatial width of the MAF has to be carefully chosen to yield a good compromise between improvement of SNR and reduction of spatial resolution. Obviously, a complete satisfactory choice regarding the degree of smoothing is not always possible. Especially in dynamic studies, in single gates of a respiratory-gated study, and in scans of obese patients SNR can be very low initially and a good SNR can only be obtained by substantially compromising spatial resolution.

The problem arises since the MAF filters indiscriminately smooth over noise-induced intensity fluctuations in homogeneous areas as well as over real intensity differences ("edges") in the image.

Therefore, locally adaptive image filters, which are able to detect and maintain edges while still smoothing over noise-induced (small scale, low intensity) fluctuations, could be an interesting alternative to MAF filtering. We have investigated a special example of this type of filters, the so-called bilateral filter (BF). Such filters are typically used in 2D image processing and were first introduced by Tomasi and Manduchi [1]. BF have already been used in medical imaging, notably in MRI [2-4] and ultrasound imaging [5,6]. Their potential usefulness has also been recognised in the field of Nuclear Medicine [7], and bilateral filtering has been proposed for improvement of iterative image reconstruction in PET [8]. However, to the best of our knowledge, performance of bilateral filtering in the PET image domain has not yet been systematically evaluated.

In this study, we investigated bilateral filtering of PET image data with respect to its effect on the SNR, the spatial resolution and, especially, the quantification of tracer uptake with the maximum of the SUV in the respective lesion, SUV_max_. Results were compared to those obtained after MAF filtering of the same data.

## Materials and methods

### The BF

A BF *W *consists of a product of two separate filters, one acting in the spatial domain (*W*_S_), one acting in the intensity domain (*W*_I_). Choosing Gaussian shapes for both components [9], the BF is given as:

(1) $$W\left(m,n\right)=W_{\text{S}}\left(P_{m}-P_{n}\right)\cdot W_{\text{I}}\left(I_{m}-I_{n}\right)=\underset{\text{spatial domain}}{\underset{︸}{\exp\left(-\frac{{\left(P_{m}-P_{n}\right)}^{2}}{2\sigma_{\text{S}}^{2}}\right)}}\cdot \underset{\text{intensity domain}}{\underset{︸}{\exp\left(-\frac{{\left(I_{m}-I_{n}\right)}^{2}}{2\sigma_{\text{I}}^{2}}\right)}},$$

where *m *is the index of the currently considered target voxel at position *P_m _*with intensity *I_m_*, and *n *enumerates the neighbouring voxels. The filter width of both components is controlled by the two free parameters *σ*_S _and *σ*_I _representing the respective standard deviations of the Gaussians. Filtering of any target voxel *m *is obtained as usual by replacing its current intensity value *I_m _*with

(2) $$\frac{\sum_{n}W\left(m,n\right)\cdot I_{n}}{\sum_{n}W\left(m,n\right)}$$

where the sum runs over all neighbouring voxels *n *with intensity *I_n _*covered by the chosen filter width. In contrast to the spatial part of the filter alone, the weighting assigned to a certain voxel *n *does not only depend on the spatial distance to the target voxel, but also on the differences of their respective intensities. This intensity-dependent part *W*_I _can be viewed as modulating the given spatial part *W*_S _in such a way that the resulting BF has a different shape for each target voxel *m*. The filter has two free parameters, namely the standard deviations *σ*_S _and *σ*_I_. These parameters, or the corresponding full widths at half maximum Γ (Γ ≈ 2.35 *σ*), define which distances to the target voxel are considered large: "distant" voxels (in space *or *intensity) are not contributing significantly to the averaging process. Only "close" voxels (in space *and *intensity) are effective in the averaging process in Equation 2.

Consequently, to preserve the edges of a certain target structure in the image data, the width of the intensity-dependent part of the filter (controlled by the parameter Γ_I_) has to be comparable to (or smaller than) the intensity difference between that target and its background.

**Figure 1:**
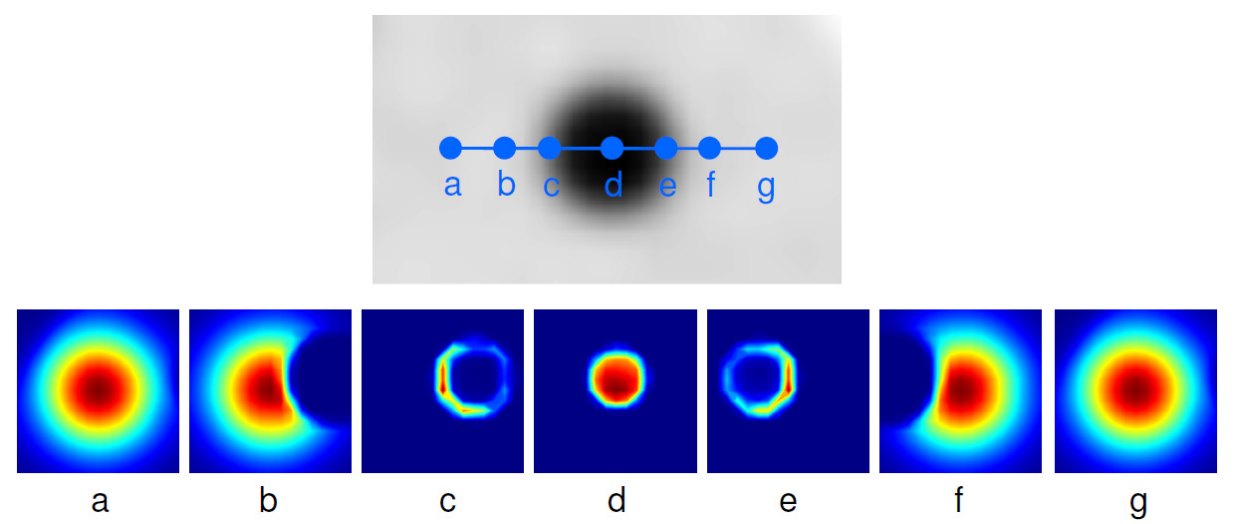
**Illustration of locally adaptive behaviour of BF**. The b/w image (top) shows the original image (hot sphere plus warm background). The blue dots denote selected positions (labelled a-g) for which the 2D BF kernel is computed and shown as colour-coded image (small weights: blue, large weights: red) at the bottom. Note how the kernel adjusts itself to the selected target position, including only neighbouring pixels of similar intensity in the averaging procedure.

If suitable parameters Γ_M_, Γ_I _are used, only nearby voxels with intensities close to that of the target voxel will contribute to the averaging. It is exactly this behaviour which leads to the edge preserving properties of BF. Figure 1 illustrates the locally adaptive nature and edge preservation mechanism of the BF in the 2D case of a single slice from a phantom measurement of a hot sphere plus warm background. The BF kernel is shown for seven selected positions along a (slightly off centre) cross section through the sphere. Note, how the kernel adjusts its shape while approaching and traversing the object. At each position, the intensity dependence of the filter ensures that pixels whose intensities differ sufficiently from the intensity of the target pixel are effectively excluded from the averaging process. Immediately at the object edge the averaging is more or less switched off completely (or rather: reduced to inclusion of pixels along the circumference), thus preserving a sharp object boundary.

### Patient group, data acquisition and data processing

The patient group included ten patients with head and neck cancer (nine men and one woman, mean age 56 ± 7.3 years) which had been scheduled for tumour staging with whole-body FDG-PET.

The PET scans were performed with an ECAT EXACT HR^+ ^(Siemens, Erlangen) using a routine acquisition protocol (2D acquisition, 8 min emission, 4 min transmission per bed position). Data acquisition started 1 h after injection (290-330 MBq FDG). Tomographic images were reconstructed from the projection data using the standard attenuation-weighted OSEM reconstruction provided with the system (6 iterations, 16 subsets, Gaussian filter with FWHM: Γ_0 _= 4 mm). In the following, these images are referred to as original images. The data were postprocessed with a Gaussian MAF (FWHM: Γ_M _= 7 mm) and BF (parameters: Γ_S _= 9 mm, Γ_I _= 2.5 SUV), respectively. The BF parameters were chosen in such a way that a comparable SNR is obtained in the filtered data with BF and MAF, respectively. SNR was quantified as described in the following section. Thirty-four lesions (volumes of about 1-100 mL) were analysed with respect to their SUV_max _values in the original as well as in the MAF and BF-filtered images. MAF and BF filtering was performed with the software ROVER (ABX GmbH, Radeberg, Germany).

### Image analysis

In the following, we use the expression SNR = *μ/σ *for computing the SNR, where *σ *is the standard deviation of the voxel intensities in a homogeneous ROI and *μ *is the corresponding mean value. Furthermore, the *noise level *is defined as the inverse of the SNR (i.e. as the ratio *σ*/*μ*).

**Figure 2:**
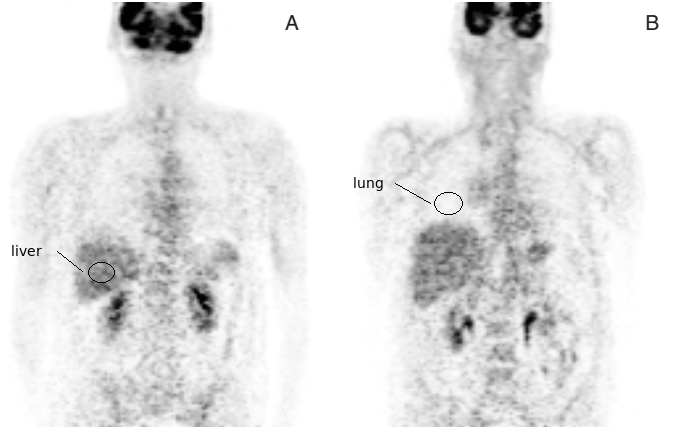
**Example of ROIs in liver **(A) **and lung **(B) **used for the determination of the noise level**.

To estimate the noise level in the image data, two spherical 3D ROIs were positioned in approximately homogeneous central regions of the liver (ROI volume: 33 mL) and the lung (ROI volume: 27 mL) as indicated in Figure 2. ROI position was chosen identical in the original and filtered image data. For each study the noise level was then determined in the original image as well as in the MAF and BF-filtered data. For each lesion the SUV_max _was determined in the original images as well as in the filtered images. Lesion volumes were derived by manual delineation of the lesions in the MAF processed data (which were preferred to the original image data by the evaluating physician) using the software ROVER (ABX GmbH, Radeberg, Germany). This software was also used for further ROI-based data analysis.

### Phantom measurement

To estimate the spatial resolution of the differently processed image data and to test the stability of BF, we performed a measurement with a water-filled cylinder phantom containing six spherical inserts (volume range 0.5-26.5 mL) at a target to background ratio of 7. The phantom data were acquired in list mode using an in-house solution [10]. This allowed retrospective variation of the effective acquisition time for generation of image data with different noise levels. Two different noise levels (as measured in a large ROI remote from the sphere inserts) where evaluated: 28%, which is comparable to the noise level in the liver encountered in our patient data, and a distinctly higher noise level of 38%. The reconstructed phantom data were then postprocessed with MAF and BF in the same way as the patient data but using three different values for Γ_I _(2.0, 2.5 and 3.0 SUV, respectively).

The spatial resolution of the phantom data was determined according to the method described in full detail in [11]: first, the image data for each sphere (plus some neighbourhood) were expressed in the form *A*(*r_n_*), where *A *is activity concentration, *n *enumerates the included voxels and *r_n _*is the distance of the respective voxel to the centre of the sphere. *A*(*r_n_*) thus represents the measured radial activity profile across the considered sphere averaged over all directions. The spatial resolution of the imaged sphere was then determined by least squares fitting the analytical profile formula *F*(*r*)--resulting from convolution of an isotropic Gaussian point spread function with a homogeneous sphere--to the measured radial profile data *A*(*r_n_*). The recovery coefficient for each sphere was finally computed as the ratio of the fitted profile at the sphere centre, *F*(0), and the known actual activity concentration in the given sphere. The recovery coefficients obtained for the differently filtered image data were then compared.

## Results

**Figure 3:**
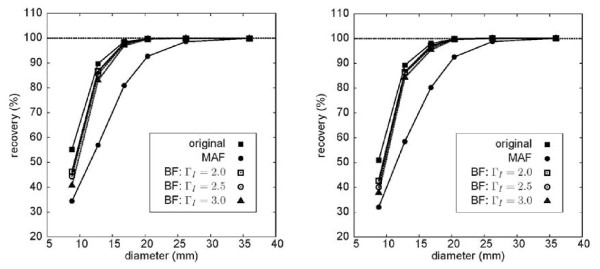
**Recovery coefficients of differently sized spheres in a warm background (sphere/background = 7) before and after filtering at two different noise levels**. Left: noise level 28%. Right: noise level 38%. MAF, moving average filter (Γ_M _= 7 mm); BF, bilateral filter (Γ_S _= 9 mm). For BF, three different settings for the width of the intensity-dependent part of the filter are used (see plot legend). A general slight decrease of signal recovery in small spheres at the higher noise level can be observed, but the increased noise does not have a specific (more pronounced) effect on BF.

**Figure 4:**
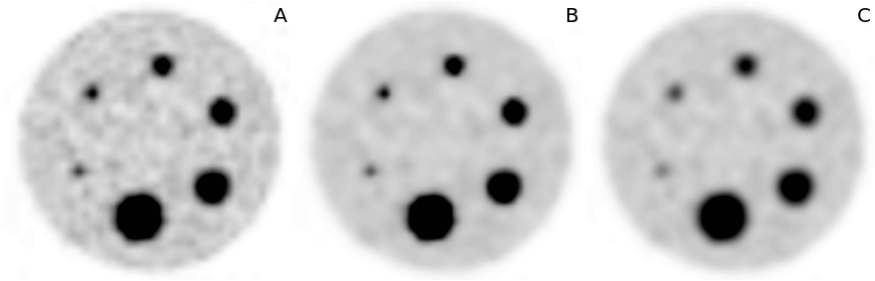
**Single transaxial slice from the phantom measurements with a background noise level of 28%**. original image **(A)**, BF-filtered data (Γ_S _= 9 mm, Γ_I _= 2.5 SUV) **(B) **and MAF-filtered data (Γ_M _= 7 mm) **(C)**.

The phantom measurements yielded the following values for the spatial resolution of the image data: (6.5 ± 0.3) mm for the original image, (6.8 ± 0.5) mm for the BF-filtered data and (9.5 ± 0.5) mm after MAF filtering. Compared to the original data, the reduction of the spatial resolution of the MAF-filtered data is obvious while BF essentially maintains the original resolution. Figure 3 shows the resulting recovery curves for both analysed noise levels. The recovery curves of the BF processed data follow essentially the curve of the original data. Only for the smallest sphere (0.47 mL), where the signal recovery in the original data is already reduced to about 50%, the recovery coefficient of the BF processed data differs substantially from that of the original data. There is only a very small difference of the performance of BF for the two investigated noise levels: signal recovery in the small spheres decreases slightly with increasing noise. However, this decrease is not specific for BF but can be equally seen in the original data and with MAF. Figure 4 shows exemplary transaxial slices of the phantom data. On the left the original image at a noise level of 28% is shown. The BF-processed data (Γ_S _= 9 mm, Γ_I _= 2.5 SUV) are shown in the middle and the MAF processed data are shown on the right. Both filters reduce the noise level significantly (from 28 to 13% (MAF) and 14% (BF), respectively). The reduced resolution of the MAF-processed data is clearly visible as is the massive drop of signal recovery in the small spheres.

**Figure 5:**
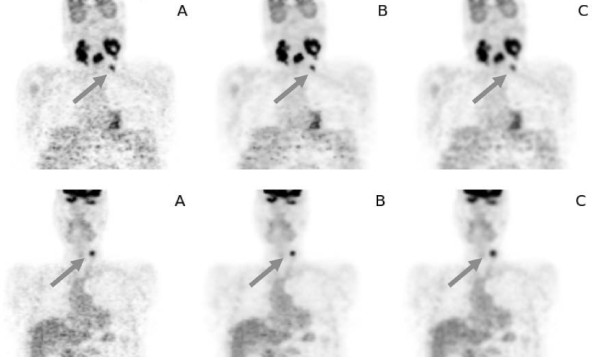
**Representative coronal sections from two patient investigation: original image**: **(A)**, BF-filtered data **(B) **and MAF-filtered data **(C)**. The SUV_max _for the small lesions marked by the arrow was 6.9 **(A)**, 6.5 **(B) **and 4.5 **(C) **at the top and 7.3 **(A)**, 6.9 **(B) **and 5.1 **(C) **at the bottom.

**Figure 6:**
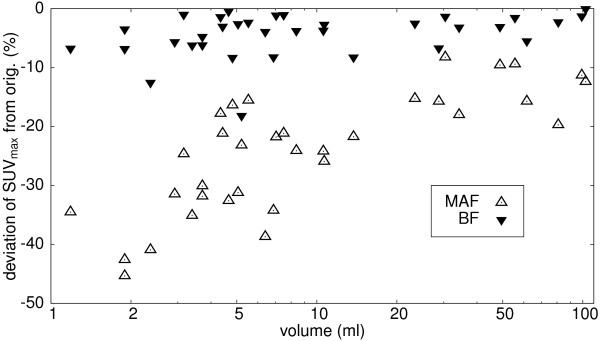
**Changes in SUV**_max _**in 34 lesions after filtering with MAF and BF, respectively**. Data are expressed as percentage of change relative to the SUV_max _values derived in the unfiltered images. Note logarithmic scale of abscissa.

Figure 5 shows representative coronal sections from two patient investigations. The original data are shown in the first column, the BF-filtered data in the middle and the MAF-filtered data on the right. The improvement of SNR after filtering is obvious as are the differences in spatial resolution after MAF and BF filtering, respectively. Table 1 shows derived noise levels for all data sets before and after filtering with MAF and BF, respectively. The noise level in the original images was (30.0 ± 6.9)% in the liver and (36.2 ± 6.2)% in the lung. After filtering noise levels were reduced to (15.7 ± 4.1)% (MAF) and (18.9 ± 3.7)% (BF) in the liver and to (20.1 ± 4.1)% (MAF) and (17.8 ± 3.7)% (BF) in the lung, i.e. with the chosen filter parameters both filters reduced the noise by about a factor of two in comparison to the original data. The SUV_max _evaluation for all 34 lesions in this patient group is shown in Figure 6. The changes in SUV_max _after filtering with MAF and BF, respectively, are plotted against the lesion volume (using a logarithmic scale for the latter to better differentiate small lesions). The reduction in SUV_max _is below 10% in 32 out of 34 lesions for BF. The two remaining lesions exhibited a very small elevation of target over background (Δ SUV ≈ 1.5), which reduces the ability of BF to differentiate between noise and true signal, see Discussion. The SUV reduction is much larger for MAF than for BF. Overall, a SUV_max _reduction of (4.6 ± 3.7)% (range 0-18%) for BF and (24.1 ± 9.9)% (range 9.2-44.5%) for MAF is observed in comparison to the unfiltered data. The reduction of the SUV_max _in the MAF data is clearly size dependent and becomes substantial for small lesions, while there is no systematic size dependency of the reduction in the BF data. Table 2 shows the derived SUV_max _for all investigated lesions.

**Table 1:** Observed relative noise level (%) in the liver and in the lung

	Study	# 1	# 2	# 3	# 4	# 5	# 6	# 7	# 8	# 9	# 10	**Mean ± Std.Dev**.
Liver	orig.	37	31	30	29	19	22	23	34	40	35	30.0 ± 6.9
	MAF	19	17	13	16	9	11	11	19	21	21	15.7 ± 4.1
	BF	23	19	13	18	11	15	11	25	28	26	18.9 ± 3.7
Lung	orig.	31	33	43	33	32	30	32	42	38	48	36.2 ± 6.2
	MAF	15	17	24	18	19	17	18	21	24	28	20.1 ± 4.1
	BF	13	14	21	16	17	16	16	19	21	25	17.8 ± 3.7

**Table 2:** SUV**_max _**in the original images and after image filtering for all investigated lesions

Lesion	Volume (mL)	**SUV_max _orig**.	SUV_max _MAF	SUV_max _BF
1	1.2	6.3	4.1	5.9
2	1.9	5.0	2.8	4.7
3	1.9	5.8	3.3	5.6
4	2.4	4.0	2.3	3.5
5	2.9	5.4	3.7	5.1
6	3.2	23	17	22
7	3.4	6.9	4.5	6.5
8	3.7	7.0	4.8	6.6
9	3.7	7.3	5.1	6.9
10	4.3	17	14	17
11	4.4	9.8	7.7	9.5
12	4.7	17	11	16
13	4.8	6.8	5.6	6.2
14	5.1	13	8.8	12
15	5.2	3.3	2.5	2.7
16	5.5	18	15	17
17	6.4	5.8	3.6	5.6
18	6.9	6.8	4.5	6.2
19	7.0	20	15	20
20	7.5	20	16	20
21	8.4	8.4	6.3	8.0
22	11	13	9.5	12
23	11	12	9.1	12
24	14	6.8	5.4	6.3
25	23	18	15	17
26	29	8.7	7.4	8.1
27	30	21	19	20
28	34	11	9.3	11
29	49	17	16	17
30	56	27	24	27
31	62	10	8.4	9.5
32	81	14	11	14
33	99	16	14	16
34	103	26	23	26

## Discussion

Bilateral filtering for edge-preserving noise reduction has been first proposed by Tomasi and Manduchi [1]. Its principal virtues have widely been recognised in 2D image processing [9,12,13] and, more recently, in medical imaging as well [2-6], but applications in Nuclear Medicine in general and PET in particular seem to have been missing up to now (we have found only one exception [14], where BF was mentioned as a preprocessing step for a new image segmentation algorithm).

In this study, we have investigated the suitability of bilateral filtering for postprocessing of PET image data. In this first assessment, we have focused on the influence of bilateral filtering on SUV_max _quantitation in FDG PET of head and neck cancer patients. This parameter is of substantial relevance in quantitative assessment of focal lesions in oncological PET in general.

The main result of this investigation is the fact that bilateral filtering with a suitable fixed choice of filter parameters is indeed able to provide a relevant improvement of the SNR (by about a factor of two) without significantly compromising the spatial resolution of hot focal lesions in comparison to the original data: in phantom measurements we found only a negligible reduction of resolution with BF by about 5%. To achieve the same degree of noise reduction with standard moving average filtering, however, one has to accept a decrease in spatial resolution of nearly 50%. With conventional smoothing filters one always faces this well-known trade-off between noise reduction and resolution loss. In clinical routine the standard procedure is to integrate a certain degree of smoothing either directly into the image reconstruction workflow or to perform the required image smoothing as a postprocessing step. Complete omission of image smoothing usually is not an option, since too high image noise would mask relevant low intensity lesions and generally hampers the diagnostic evaluation of the images by the physician. Given this necessity for improvement of the SNR, our results indicate that bilateral filtering is a superior alternative to the standard smoothing filters.

Evaluation of the performance of bilateral filtering in a group of patients from clinical routine implies that our investigation does suffer from the absence of a true gold standard: we only compared SUV_max _values after filtering (with MAF and BF, respectively) with those obtained in the original data, which in turn are not necessarily guaranteed to be correct. Regarding MAF filtering, our results confirm the well-known fact that image smoothing with MAF filters reduces spatial resolution, therefore increases partial volume effects and consequently compromises quantification accuracy, especially in small lesions (and ultimately prevents detection of lesions near the resolution limit). Quantification errors in small lesions (relative to the values derived in the original data) easily approached 30-40% in our investigation when using MAF, see Figure 6. After BF filtering, however, we observed in 32 out of 34 lesions only a slight reduction of SUV_max _(mean ± std. dev.: (4.6 ± 3.7)%) but this small reduction of the SUV_max _is probably explainable by the achieved noise reduction in the interior of the lesion which necessarily reduces the value of the maximum voxel. This effect decreases the known bias of SUV_max _values [15] caused by selecting the single hottest voxel of the lesion. In this sense it is very well possible that the BF filtered SUV_max _value is a better estimate of the true SUV than the value derived from the original data, but verification of this conjecture would of course require further investigation. This contrasts to the consequences of MAF filtering where the indiscriminate reduction of spatial resolution massively decreases signal recovery in small lesions. This is the reason for the strong size dependence of the SUV_max _reduction after MAF filtering demonstrated in Figure 6. The effect can also be appreciated in Figure 5 where the blurring and concomitant signal reduction in the small lesions is obvious (quantitatively it amounts to 35% (top) and 30% (bottom) in these examples). Moreover, reduction of spatial resolution and blurring of the object boundaries by MAF filtering does of course also affect quantitation of larger lesions where SUV_max _alone often is not a suitable measure and parameters like lesion volume and SUV_mean _are more relevant.

In this study, we have chosen a Gaussian BF with fixed parameters Γ_S _= 9 mm and Γ_I _= 2.5 SUV. As with any filter, this choice will not be ideal for all applications but it seems quite well suited for the image characteristics (noise level, target to background contrast, spatial resolution) usually found in oncological PET. Our phantom studies, moreover, have shown that results are neither sensitive to a variation of Γ_I _between values of 2 and 3 SUV nor to a modest variation in noise level. (The slight decrease of signal recovery in small spheres is not specific to BF but is equally present in the original data and with MAF and implies a slightly reduced reconstructed resolution at the higher noise level which would be a limitation of the iterative image reconstruction.) Nevertheless, the performance of BF in other studies than head and neck is not covered by this study and the suitability of the chosen filter parameters in a different setting would have to be checked in each case.

The relatively large spatial width of the filter is only effective in overall homogeneous image regions, i.e. regions where intensity variations are well below Γ_I_: the combined filter weight *W*(*m*, *n*) already decreases to 50% of the value defined by the spatial part *W*_S_(*P_m _*- *P_n_*) of the filter alone when the intensity difference relative to the target voxel becomes equal to 0.5 · Γ_I_. Loosely speaking this reduces the "effective width" of the complete filter for such neighbouring voxels by a factor of two. The value chosen for Γ_I _thus discriminates in a smooth manner between intensity differences (relative to the target voxel) which are considered as "noise" or "signal". However, this intensity-based discrimination will only work as long as the local target to background contrast is not too small. This limitation is exemplified by the two lesions where the reduction of the SUV_max _exceeded 10% (see Figure 6). These lesions exhibited a low target to background contrast of about 2 and, more important, only a small SUV elevation above background of about 1.5 SUV units. For these lesions, the discrimination between "noise" and "signal" works not very well and the BF produces results for these lesions which are similar to (although not as bad as) those obtained by MAF filtering. This reduced performance of the BF in small lesions exhibiting at the same time only a small elevation above the surrounding background could theoretically be avoided by choosing a smaller value of Γ_I_. However, if Γ_I _is reduced too much, even noise-induced intensity changes would be interpreted as "edges" and the filter would effectively be switched off. The chosen parameter value thus is always a compromise between edge preservation and noise reduction. It should be noted, however, that for one, performance even in these cases is better (signal recovery still higher) than for a MAF filter of comparable noise reduction and, moreover, that the problem actually only arises for lesions which at the given SNR are hardly identifiable at all in the data. On the other hand, MAF filtering of small lesions always causes substantial problems, independent of their target to background ratios.

In view of the fact that the SUV_max _is still the most often used quantitative PET parameter for therapy response assessment [16] its reduction in the MAF-filtered data is especially problematic. In this setting (therapy response control), the SUV_max _might be assessed several times before, during and after therapy. Only if the lesion does not change in size MAF-filtered data will provide correct information regarding fractional changes of tracer uptake since in this case the systematic signal reduction due to the (increased) recovery effect will be constant. This is no longer true, however, if the lesions' size decreases under therapy. In such a case the increased recovery effects after MAF filtering can lead to a spurious decrease of the derived SUV_max _values which does not correspond to an actual reduction of tracer uptake. While these effects do always occur near the limit of the given spatial resolution, they are obviously aggravated by the use of MAF filtering. The edge-preserving properties of the BF thus constitute a major advantage over MAF filtering. We therefore believe that it is worthwhile to more thoroughly evaluate this promising tool in the future to determine its full potential as well as its limitations for different types of studies and tomographs.

## Conclusion

Bilateral filtering exhibits superior properties in comparison to the smoothing filters routinely applied for noise reduction in PET. Bilateral filtering allows to increase the SNR of PET image data while preserving spatial resolution at object boundaries, thus maintaining the quantitative accuracy of SUV_max _values even in small lesions. Therefore, it seems worthwhile to investigate more thoroughly the potential of this filter as a replacement of the standard smoothing filters and for improving the image quality of diagnostic PET.

## Competing interests

The authors declare that they have no competing interests.

## Authors' contributions

FH implemented the final version of the 3D BF, performed part of the data analysis and is the main author of the manuscript. JL performed the phantom measurements and the image reconstruction. BBB selected the patient studies and performed the lesion delineation. LO performed part of the data analysis. JS and JK provided intellectual input and reviewed the manuscript. JVDH implemented a prototype of the 3D BF for initial testing and wrote part of the manuscript. All authors read and approved the final manuscript.

## References

[B1] Tomasi C Manduchi R Bilateral Filtering for Gray and Color Images Proc IEEE Int Conf Comput Vis 1998 0 839

[B2] Walker S Miller D Tanabe J Bilateral spatial filtering: Refining methods for localizing brain activation in the presence of parenchymal abnormalities NeuroImage 2006 33 2 564 569 10.1016/j.neuroimage.2006.06.051 16942890

[B3] Kosior J Kosior R Frayne R Robust dynamic susceptibility contrast MR perfusion using 4D nonlinear noise filters J Magn Reson Imaging 2007 26 6 1514 1522 10.1002/jmri.21219 17968968

[B4] Xie J Heng P Shah M Image diffusion using saliency bilateral filter IEEE Trans Inf Technol Biomed 2008 12 6 768 771 19000957 10.1109/TITB.2008.926462

[B5] Balocco S Gatta C Pujol O Mauri J Radeva P SRBF: Speckle Reducing Bilateral Filtering Ultrasound Med Biol 2010 36 8 1353 1363 10.1016/j.ultrasmedbio.2010.05.007 20691924

[B6] Tang J Guo S Sun Q Deng Y Zhou D Speckle reducing bilateral filter for cattle follicle segmentation BMC genomics 2010 11 Suppl 2 S9 10.1186/1471-2164-11-S2-S9 21047390 PMC2975414

[B7] Lee J Geets X Grégoire V Bol A Edge-preserving filtering of images with low photon counts IEEE Trans Pattern Anal Mach Intell 2008 1014 1027 18421107 10.1109/TPAMI.2008.16

[B8] Zhou J Zhu H Shu H Luo L A generalized diffusion based inter-iteration nonlinear bilateral filtering scheme for PET image reconstruction Comput Med Imaging Graph 2007 31 6 447 57 10.1016/j.compmedimag.2007.04.003 17574817

[B9] Paris S Kornprobst P Tumblin J Durand F Bilateral Filtering: Theory and Applications Foundations and Trends in Computer Graphics and Vision 2008 4 1 75 10.1561/0600000020

[B10] Langner J Bühler P Just U Pötzsch C Will E van den Hoff J Optimized list-mode acquisition and data processing procedures for ACS2 based PET systems Z Med Phys 2006 16 75 82 16696373 10.1078/0939-3889-00294

[B11] Hofheinz F Dittrich S Pötzsch C van den Hoff J Effects of cold sphere walls in PET phantom measurements on the volume reproducing threshold Phys Med Biol 2010 55 4 1099 113 10.1088/0031-9155/55/4/013 20107246

[B12] Elad M On the origin of the bilateral filter and ways to improve it IEEE Trans Image Process 2002 11 10 1141 1151 10.1109/TIP.2002.801126 18249686

[B13] Barash D A fundamental relationship between bilateral filtering, adaptive smoothing, and the nonlinear diffusion equation IEEE Trans Pattern Anal Mach Intell 2002 844 847

[B14] Geets X Lee J Bol A Lonneux M Gregoire V A gradient-based method for segmenting FDG-PET images: methodology and validation Eur J Nucl Med Mol Imaging 2007 34 9 1427 38 10.1007/s00259-006-0363-4 17431616

[B15] Boellaard R Krak N Hoekstra O Lammertsma A Effects of noise, image resolution, and ROI definition on the accuracy of standard uptake values: a simulation study J Nucl Med 2004 45 9 1519 27 15347719

[B16] Wahl R Jacene H Kasamon Y Lodge M From RECIST to PERCIST: Evolving Considerations for PET response criteria in solid tumors J Nucl Med 2009 50 Suppl 1 122S 50S 19403881 10.2967/jnumed.108.057307 PMC2755245

